# Liquid biopsy in the assessment of microRNAs in oral squamous cell carcinoma: A systematic review

**DOI:** 10.4317/jced.59736

**Published:** 2022-10-01

**Authors:** Gaspare Palaia, Roberto Pippi, Federica Rocchetti, Martina Caputo, Federica Macali, Ahmed Mohsen, Alessandro Del Vecchio, Gianluca Tenore, Umberto Romeo

**Affiliations:** 1 Department of Oral Sciences and Maxillofacial Surgery, Sapienza University of Rome

## Abstract

**Background:**

The identification of non-invasive biomarkers from biological fluids collected by liquid biopsy provides new horizons for individualized therapeutic strategies and improves clinical decision-making in OSCC patients. Circulating microRNAs have emerged as biomarkers that may reflect not only the existence of cancer, but also the dynamic, malignant potential, and drug resistance of tumors. The aim of the systematic review is to evaluate and summarize the results of the published studies regarding the use of microRNAs as biomarkers for OSCC.

**Material and Methods:**

A literature search was conducted on PubMed, Scopus, Web of Science, and Cochrane databases till November 2020. A total of 34 studies met the inclusion criteria and were therefore subjected to quality assessment. Each study was subjected to data extraction including; patient characteristics, type of fluid sample (whole blood, plasma, serum, or saliva), molecular analysis method, specific dysregulated microRNA, and microRNA expression pattern.

**Results:**

The analysis showed that 57 microRNAs of liquid biopsy samples of four different fluids (whole blood, serum, plasma, and saliva) were analyzed. The prognostic and therapeutic significance of these microRNAs were suggested by several studies; where 41 microRNAs were upregulated while 16 were downregulated.

**Conclusions:**

Scientific evidence supports the interest in the use of microRNAs in the diagnosis and prognosis in OSCC patients; however, further studies in a larger cohort of patients are mandatory to introduce liquid biopsy in the routine clinical practice for the OSCC management.

** Key words:**Biomarkers, liquid biopsy, microRNA, oral squamous cell carcinoma, systematic review.

## Introduction

Oral squamous cell carcinoma (OSCC) represents the most common histo-type of head and neck cancers, representing 90% of diagnosed oral malignancies (1). Despite the advancements in prevention, diagnosis, and management of cancer, OSCC remains a serious condition in global public health with 300,000 new diagnosed cases a year and causing 145,000 deaths per year (2).

The etiology of OSCC is not still comprehensively understood. It may be multifactorial, where a combination of several genetic alterations and environmental risk factors may play a role, including tobacco and alcohol consumption, human papillomavirus (HPV) infection, and chronic mechanical trauma. OSCC can be evolved from oral potentially malignant disorders, such as oral leukoplakia, erythroplakia, lichen planus, smoking hyperkeratosis, and submucous fibrosis, or from normal epithelium with genetically altered keratinocytes.

To date, histopathological analysis of a tissue biopsy is the gold standard for the diagnosis of OSCC. Some limitations can be considered of tissue biopsy; including that it is an invasive, costly, and potentially harmful procedure; furthermore, it is temporally and spatially limited, where it provides a snapshot of a single region of a heterogeneous tumor.

Nowadays, cancer research is encouraging to find less invasive methods to provide a more comprehensive view of the cancer profile and to easily monitor its evolution and therapeutic response. In this regard, liquid biopsy has been recently proposed as a method for diagnosis, prognosis, and follow-up of OSCCs.

The liquid biopsy involves the study and analysis of body fluids; such as blood, saliva, urine, seminal plasma and cerebrospinal fluid (3).

In this method, include the analysis of circulating tumor cells (CTCs), circulating cell-free DNA (cfDNA) or circulating tumor DNA (ctDNA), extra-cellular vehicles (EVs) and microRNAs (miRNAs) (4).

In case of confirmation of the reliability of this method for the diagnosis, prognosis, and follow-up of OSCCs, it may provide the dynamic observation of cancer evolution and for understanding temporal and spatial heterogeneity of tumors. Furthermore, it can also be used to detect early onset of therapy resistance, residual disease and recurrence.

miRNAs are a large family of single-stranded non-coding RNA that includes 19-25 nucleotides, regulating the gene expression at the post-transcriptional level.

miRNAs play a crucial role in cell cycle regulation, differentiation, apoptosis and migration. During carcinogenesis, some miRNAs are upregulated and some are downregulated, so any change in the expression of miRNAs can cause tumor suppression or act as carcinogens (5).

Circulating miRNAs are extremely sTable under harsh conditions, such as high temperature, extreme pH, and in the presence of RNAs activity, suggesting their potential role as biomarkers for cancer diagnosis (6).

Several studies reported an altered expression of blood and salivary miRNAs in different tumor types, including colon, lung, ovarian and breast cancers (7-10); however, there is not consensus regarding the clinical value of miRNAs in liquid biopsy for the management of OSCC (11).

The aim of this systematic review was to evaluate and summarize the results of the published studies regarding the use of miRNAs as biomarkers for OSCC.

## Material and Methods

This study was performed according to the PRISMA (Preferred Reporting Items for Systematic Reviews and Meta-analysis) guidelines (12). The protocol was registered at PROSPERO “the International Prospective Register of Systematic Reviews” with a reference Number CRD42020222723.

The focus question was: “Do circulating miRNAs play a role as biomarkers in OSCC?”

-Eligibility Criteria

The inclusion criteria were: 1) studies with significantly dysregulated miRNAs from body fluids between OSCC patients and healthy controls; 2) studies which analyzed the presence of dysregulated miRNA in at least one of the body fluids among blood, serum, plasma, or saliva; 3) studies involving humans; 4) studies published in peer-reviewed journal; 5) and studies in English language.

The Exclusion criteria were: 1) case-reports, case-series, conference proceedings, letters to the editor, short communications, systematic reviews; 2) *in vitro* or *in vivo* animal studies.

-Search Strategy

An electronic search was conducted on PubMed, Scopus, Web of Science, and Cochrane Library databases till 25th November 2020. The following combination of keywords were used with the Boolean term “AND” and “OR”: (“microRNA” OR “miRNA”) AND (“squamous cell carcinoma” OR “squamous cells carcinoma” OR “squamous cell carcinomas” OR “squamous cells carcinomas”) AND (“oral” OR “mouth” OR “tongue” OR “gingiva” OR “buccal”). In addition, a manual search of references listed in each included study was performed, to avoid losing potential studies.

-Study Selection

The study selection was carried out in two stages. First, two reviewers (M.C. and F.R.) identified independently the possible eligible studies by screening the titles and abstracts of the resulted studies based on the abovementioned inclusion and exclusion criteria. Then, a full-text read was carried out independently by the same two reviewers for each identified study in the first stage. In case of disagreement between the two reviewers, the final decision for the inclusion of a study was taken by consensus discussion by the inclusion of a third reviewer (G.P.).

-Data-Collection and Synthesis Process

The two investigators (M.C. and F.R.) independently assessed each eligible article, extracted data using a pre-established form using a Microsoft Excel spreadsheet (Microsoft Corp. Redmond, WA, USA). Discordant judgments were resolved by consensus discussion with the third investigator (G.P.).

The following information were extracted from each study: (i) authors, (ii) country, (iii) year of publication, (iv) sample size, (v) gender, (vi) mean age, (vii) TNM staging, (viii) histological grading, (ix) type of fluid sample (blood, plasma, serum, or saliva), molecular analysis method, (x) specific dysregulated miRNA, and (xi) miRNA expression pattern.

In case of missing or incomplete data, an email was sent to the authors to obtain more detailed information; in case of missing answer, the article was excluded.

-Reporting Quality and Risk of Bias Assessments

The quality assessment of the included studies was carried out using the SIGN “Scottish Intercollegiate Guidelines Network” methodology checklist for case-control studies. The SIGN checklist consisted of 11 statements to evaluate the risk of bias of the internal validity across 6 domains; groups comparability, differentiation, assessor blinding, outcome measures, confounding, statistical analysis. The scores for each statement were; “yes” (low risk) or “no” (high risk). In case of lack of details, the score “can’t say (moderate risk)” was given.

After the piloted test of the checklist to confirm its compatibility with this review, the reviewers decided that grouped domains “groups comparability and differentiation”, that consists of more than one statement, would have got the final score “low risk” in case all responses to the statements were “yes”, and it would be “high risk” in case of presence of a negative response (no) or the presence of two or more than of “can’t say” responses. While the final score of “moderate” was given in case of the presence of one response of “can’t say” to one of the statements.

Two reviewers (F.R. and F.M.) assessed independently each included study with this checklist. In case of conflicts, a third reviewer (G.P.) was consulted for arbitration. Finally, an overall assessment was provided to each study by the reviewers with the same scores.

## Results

**Figure 1:**
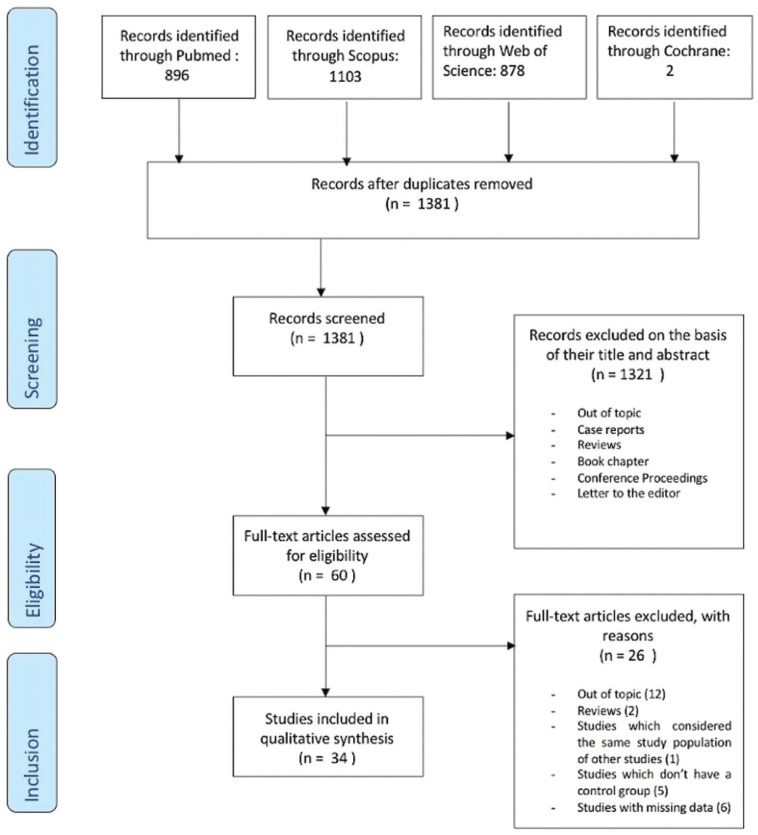
PRISMA flow diagram showing the articles included in the final analysis (34) that met the inclusion criteria.

A total of 2879 studies were resulted from the search of the electronic databases and were distributed as follows; 896 studies from PubMed, 1103 studies from Scopus, 878 studies from Web of Science, and 2 studies from Cochrane database. After the removal of duplicates, a total of 1381 articles were screened. The title and abstract screening resulted in the exclusion of 1321 studies. After the full-text screening, 26 articles were excluded due to the following reasons: out of topic (n=14); insufficient information (n=6); studies considered the same study population of other studies (n=1); studies without a control group (n=5). No disagreement regarding study eligibility was observed between the reviewers. No additional relevant studies were found by checking references and citations of the included studies. A detailed flow chart of the selection process is shown in Figure 1. Thirty-four studies met the inclusion criteria and were considered for the final analysis.

**Figure 2:**
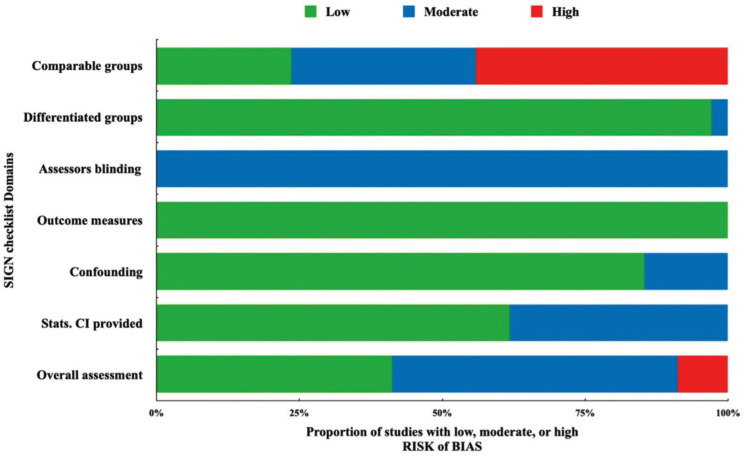
The proportion of the studies of this review with a low, moderate, or high risk of bias across the different considered domains of the SIGN methodology checklist.

In the overall assessment of the risk of bias, fourteen studies of a total of 34 studies were rated with a low risk score. The majority (17 studies) of the included studies were awarded a moderate risk score. This was because of the partial or poor identification of the population source with an incomplete demonstration of the baseline similarities and/or differences between the case and control groups. Also, because some studies didn’t provide a complete statistical analysis including the confidence interval (CI). The presence of blinding assessor in all the included studies of this review was unclear. Only 3 studies were rated with a high-risk score, this was mainly due to the incomplete reporting of the participant demographic data mainly that of the control group and the poor presentation of comparison between participants and non-participants to establish their baseline similarities or differences (Table 1,  Table 1 cont.). Figure 2 shows the proportion of studies of this review with a low, moderate, or high risk of bias across the different considered domains of the SIGN checklist.

**Table 1:**
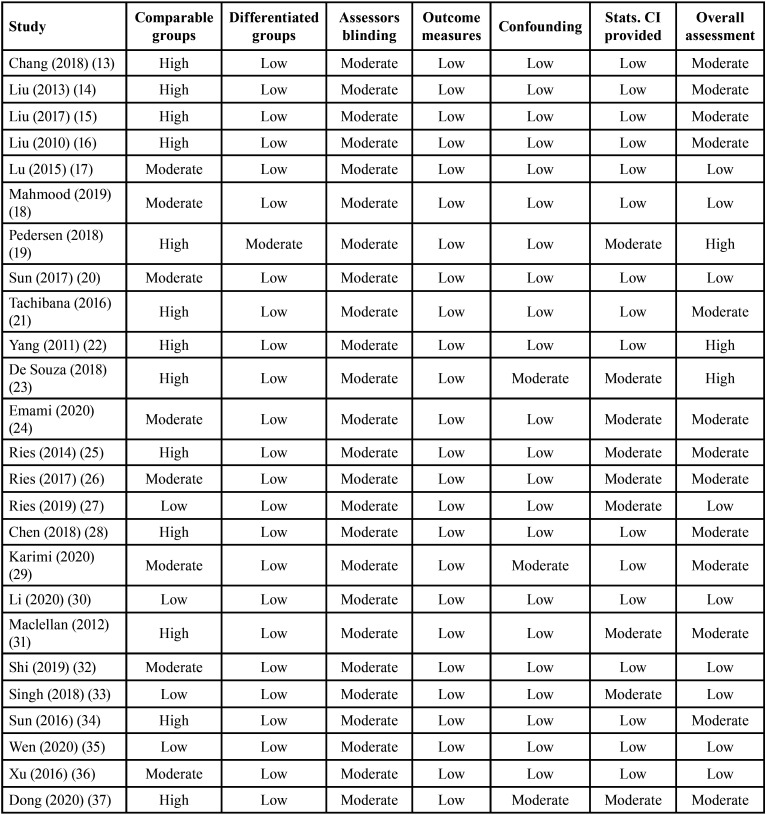
Risk of bias assessment scores of the included studies with SIGN methodology checklist for case-control studies.

**Table 1 cont.**
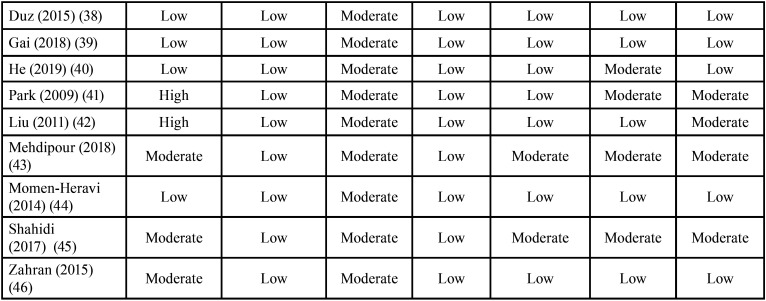
Risk of bias assessment scores of the included studies with SIGN methodology checklist for case-control studies.

The characteristics of the included studies are presented in Table 2. According to the type of liquid biopsy samples, the included studies were divided into two groups; group A consisted of 24 studies that were carried out on blood samples, and group B consisted of 9 studies that were carried out on saliva samples. One study was performed on both serum and saliva and was included in both groups. In group A, there were 5 studies that were carried out on whole blood, 10 studies only on serum, and 10 studies only on plasma. In group B, there were 10 included studies on saliva.

**Table 2:**
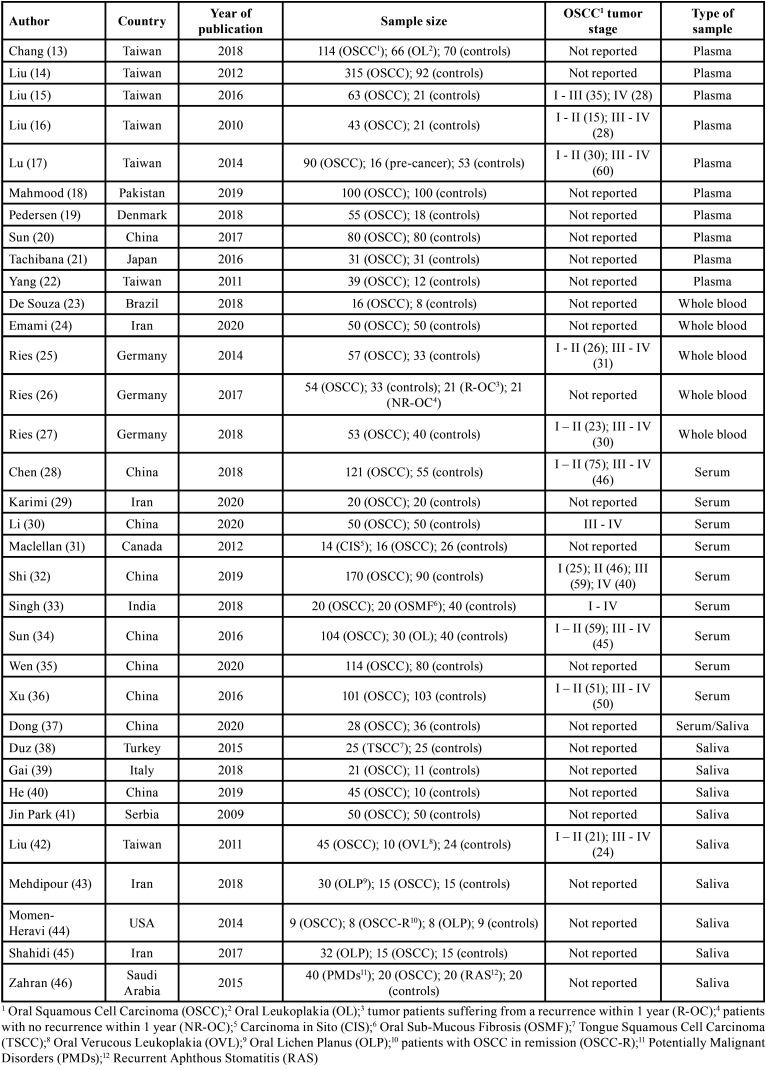
General characteristics of included studies.

-Group A features

Group A involved 25 case-control studies published from 2010 to 2020 and carried out in 10 different countries, where 8 studies in China, 6 studies in Taiwan, 3 studies in Germany, 2 studies in Iran, and 1 study in each of the following countries: Canada, Japan, Brazil, Denmark, and Pakistan. The sample size of the studies ranged from 16 to 170, and comprised a total of 2730 subjects (1606 OSCC patients and 1124 healthy control patients).

The percentage of males and females was reported in 19 of the 25 studies. Males represented 62.6% while females were 37.4%. The mean age of OSCC patients was 57 years (20 - 93 years). The percentage of alcohol consumers among OSCC patients was reported in 8 of 25 studies, and 54.1% of the studied patients were alcohol consumers. With regard to smoking, 81.5% of the studied patients were smokers.

Histological grading of OSCC was reported in 10 of the 25 studies: 28% of OSCC were well-differentiated, 12% were between well-differentiated and moderately differentiated, 24% were moderately differentiated, 4% were between moderately differentiated and poorly differentiated, and 32% were poorly differentiated. Tumor staging was reported in 12 studies: stage I (10%), stage I-II (23%), stage II (10%), stage I-III (3%), stage III (14%), stage III-IV (23%), and stage IV (17%). Eleven studies reported the presence/absence of lymph node involvement (N), of which 48% was N0, and 52% was N+. Only 4 studies reported the presence/absence of distant metastases (M), of which 15% were M+. With regard to the site of OSCC, 33% of OSCC were on the tongue, 17% on the buccal mucosa, 17% on the gingiva, 25% in other oral sites, and 8% in unspecified sites.

The control group consisted of 371 males and 184 females with a mean age of 54 years (15 - 88 years). In this group, 18.3% were smokers, and 12% were alcohol consumers.

Expression levels of single miRNA (n=34) were detected by quantitative Polymerase Chain Reaction in real-time. According to the individual miRNA expression levels, 23 miRNAs were up-regulated and 11 were down-regulated (Table 3).

**Table 3:**
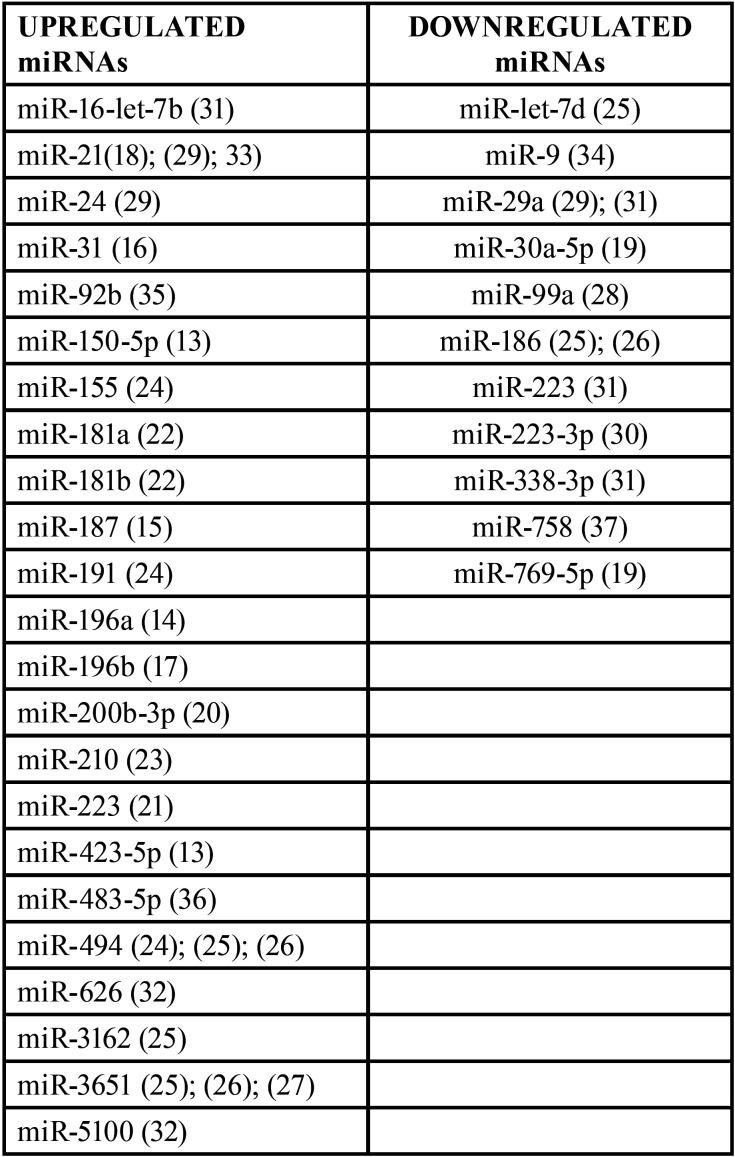
miRNAs upregulated and downregulated in group A.

-Group B features

Group B involved 10 case-control studies published from 2009 to 2020 and carried out in 8 different countries; 2 studies in Iran, 2 studies in China, and 1 study in each one of the following countries: Taiwan, USA, Turkey, Saudi Arabia, Serbia, and Italy.

The sample size of studies included in Group B ranged from 9 to 50 and comprised a total of 372 subjects (277 OSCC patients and 95 healthy control patients).

The percentage of males and females was reported in 7 of the 10 studies. Males were 65.5% and females were 34.5%. OSCC patients in Group B had a mean age of 48.8 (20 - 78 years).

The percentage of alcohol consumers among OSCC patients was reported in 3 out of the 10 studies. Alcohol consumers represented 26% of all the patients. While the percentage of smokers was reported in 5 of the 10 studies, 59% of the studied patients were smokers.

Histological grading of OSCC was reported only in 2 studies: 10% of OSCC were well differentiated, 20% were moderately differentiated, 10% were between moderately differentiated and poorly differentiated, 20% were poorly differentiated, 20% were undifferentiated or anaplastic. Tumor staging was reported only in two studies: stage I (10%), stage I-II (10%), stage II (10%), stage III (10%), stage III-IV (10%), and stage IV (10%). Only one study analyzed the lymph node involvement, of which 67% was N0 and 33% was N+. OSCC site has been reported in two studies; where 40% of OSCC was on the tongue, 20% of them were on the buccal mucosa, 20% on the gingiva, and 20% on other oral sites.

The control group involved 46 males and 23 females with a mean age of 53.5 (37-75 years). In this group, 30.5% were smokers and 9.5% were alcohol consumers.

Expression levels of single miRNA (n=27) were detected by quantitative Polymerase Chain Reaction in real-time. According to the individual miRNA expression levels, 21 miRNAs were upregulated and 6 were downregulated ( Table 4).

**Table 4:**
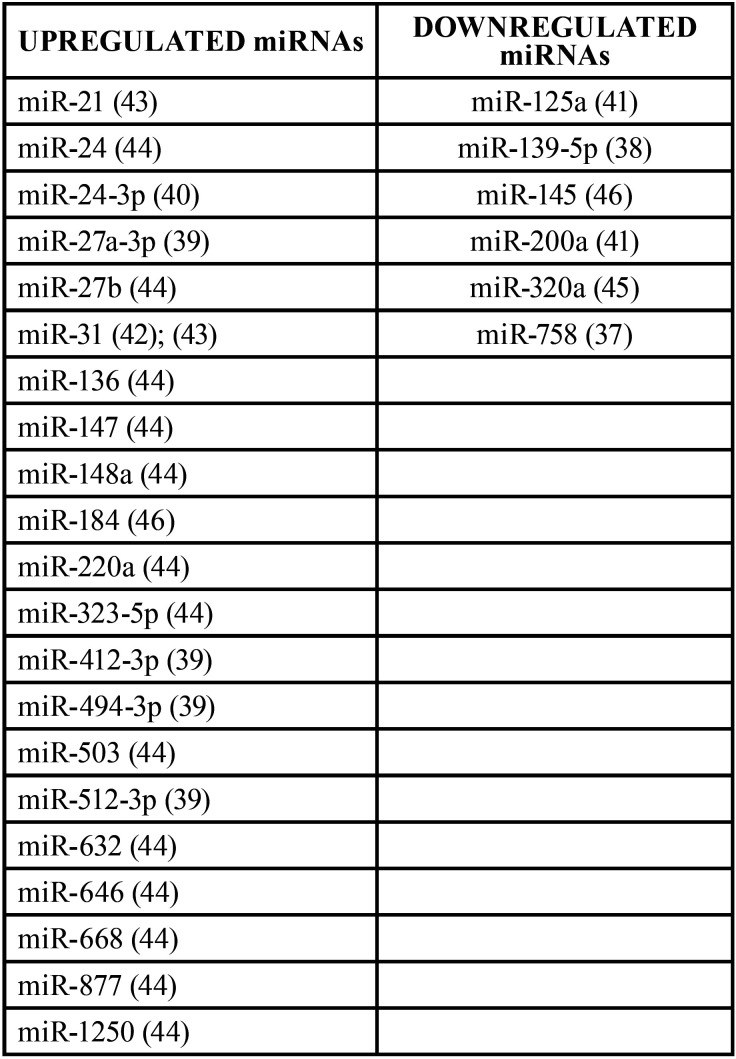
miRNAs upregulated and downregulated in group B.

## Discussion

Recently, many efforts have been made to improve OSCC diagnosis, treatment, and prognosis. However, according to GLOBOCAN, OSCC represents one of the leading causes of morbidity and mortality worldwide (2).

This primarily depends on the delay of the patient being screened and on the diagnostic delay (47). Furthermore, due to the intra-tumoral and inter-tumoral heterogeneity and the dynamic behavior of cancer with the modification of the molecular profile over time, the traditional strategies of OSCC screening are not sufficient for the effective management of this cancer (4,48).

In light of the above, the latest studies have been focused on understanding the molecular basis of OSCC, to develop more precise and personalized methodologies for early detection, prognosis, and establishment of successful therapies.

Liquid biopsy with circulating biomarkers could represent a new opportunity to understand the molecular profile and dynamic behavior of the tumor (49). There is increasing evidence that small non-coding RNAs (mainly miRNAs) may play an important role in cancer pathogenesis. In addition, miRNA obtained using liquid biopsy has recently emerged as a potential biomarker for oral cancer (50,51).

The aim of the present study was to systematically review the current findings on miRNAs in OSCC patients. The search was carried out in four of the most common databases with a focus on studies with statistically differential expression of one miRNA in one of the body fluids in patients suffering from OSCC compared to healthy control. The year of publication of the included studies ranged from 2013 to 2020. Most of the studies were conducted on plasma, serum, and saliva samples, while whole blood samples were less investigated.

All the studies showed heterogeneous sampling regarding the stage of the tumor. All the studies showed heterogeneous sampling regarding the stage of the tumor. Of note, the subgroup analysis performed by Liu *et al*. demonstrated that OSCC patients affected by early-stage (T:1–2 with N0) had significantly higher levels of miR-31 in their preoperative plasma compared to the healthy controls. Furthermore, the majority of the OSCC patients showed a decrease in plasma miR-31 levels after surgical resection (16). This would be important since the possibility to discover a biomarker in the early stages of OSCC represents a major goal for predicting future metastasis.

In addition, the potential role of micro-RNAs in predicting lymph nodal metastasis was evaluated in several studies (13,19,22,36). Chang *et al*. (13) carried out a study to identify the potential plasma miRNAs for the early detection of oral cancer evaluating that through comparing samples from healthy subjects, patients with oral leukoplakia, and OSCC patients. Three miRNAs “miR-222-3p, miR-150-5p, and miR-423-5p” were identified and differentially expressed among groups. The level of expression of the miR-423-5p and miR-222-3p was identified significantly in oral cancer tissues and was involved in tumor progression and lymph node metastasis. The authors concluded that these three plasma miRNAs may be potential biomarkers for the early detection of oral cancer and may be helpful for monitoring the malignant progression from oral leukoplakia to OSCC.

Similarly, Yang *et al*. (22) studied the correlation between the up-regulation expression of miR-181 in the tissue and plasma of OSCC patients and multiple clinical variables as a trial to understand the function of miR-181 in oral tumorigenesis. Interestingly, among different clinical variables, the authors found a correlation between the over-expression of miR-181 and lymph-node metastasis, vascular invasion, and poor survival. In addition, the functional assays revealed that the over-expression of miR-181 may have enhanced the cell migration and invasion. They concluded that the miR-181 may be considered as a putative biomarker for OSCC patients where miR-181 may regulate cell migration and eventually enhance the lymph-node metastasis.

Moreover, in the study by Xu *et al*. (36), to explore the abnormal miRNA profile in serum samples from OSCC patients and the association between the miR-483-5p expression and the patient prognosis, it was found a significant over-expression of miR-483-5p in OSCC patients and a significant correlation between it and the tumor nodal metastasis (TNM) stage and lymph node metastasis. The authors proposed that the expression levels of miR-483-5p may be considered a diagnostic and prognostic biomarker for OSCC.

Pedersen *et al*. (19), in a study to define the miRNAs in OSCC and normal oral mucosa, and to identify and validate new diagnostic miRNAs and miRNA combinations, found that miR-1307-5p, miR-671-5p, and miR-769-5p levels were differentially expressed between OSCC patients with and without lymph node metastases.

The prognostic significance of microRNAs as a biomarker was also evaluated. Liu *et al*. (14), showed a possible association between the higher level of miR-196-a and the worse prognosis of OSCC. The study by Sun *et al*. (34), showed that miR-200b-3p was significantly up-regulated in plasma of 80 OSCC patients compared with healthy controls and that high miR-200b-3p expression was associated with poor prognosis. Also, Shi *et al*. (32), demonstrated that high expression of miR-626 and miR-5100 was significantly related to poor prognosis.

Another important issue was addressed which is the possibility to predict the response to treatment. Karimi *et al*. (23) reported that miR-21 was associated with resistance to chemotherapy.

Of the included studies, only Tachibana *et al*. (21) focused on OSCCs from a single subsite of the oral cavity (gingiva) while the others did not report about subsite of the tumor or did not perform subgroup analysis to investigate possible differences.

Our review presents some limitations: firstly, the absence of meta-analysis because of the heterogeneity of the data. Secondly, studies did not have large sample size and long follow-up, which may raise questions on the reliability of the outcomes. Finally, several studies included were conducted on Asian population with only few studies involved patients from a different ethnic population.

The field of liquid biopsy has gained more interest over the last two decades. Due to their stability in a variety of body fluids, miRNAs have been shown to be promising non-invasive biomarkers for the diagnosis, prognosis, and therapeutic goals of OSCC. Further studies in a larger cohort of patients are mandatory to introduce liquid biopsy in the routine clinical practice for the OSCC management.

## References

[1] Pires FR, Ramos AB, Oliveira JB, Tavares AS, Luz PS, Santos TC (2013). Oral squamous cell carcinoma: clinicopathological features from 346 cases from a single oral pathology service during an 8-year period. J Appl Oral Sci.

[2] Ferlay J, Soerjomataram II, Dikshit R, Eser S, Mathers C, Rebelo M (2015). Cancer incidence and mortality worldwide: Sources, methods and major patterns in GLOBOCAN 2012. Int J Cancer.

[3] Arantes LMRB, De Carvalho AC, Melendez ME, Carvalho AL (2018). Serum, plasma and saliva biomarkers for head and neck cancer. Expert Rev. Mol. Diagn.

[4] Cristaldi M, Mauceri R, Di Fede O, Giuliana G, Campisi G, Panzarella V (2019). Salivary Biomarkers for Oral Squamous Cell Carcinoma Diagnosis and Follow-Up: Current Status and Perspectives. Front Physiol.

[5] Weber JA, Baxter DH, Zhang S, Huang DY, Huang KH, Lee MJ (2010). The microRNA spectrum in 12 body fluids. Clinical chemistry.

[6] Detassis S, Grasso M, Del Vescovo V, Denti MA (2017). microRNAs Make the Call in Cancer Personalized Medicine. Front Cell Dev Biol.

[7] Galamb O, Barták BK, Kalmár A, Nagy ZB, Szigeti KA, Tulassay Z (2019). Diagnostic and prognostic potential of tissue and circulating long non-coding RNAs in colorectal tumors. World J Gastroenterol.

[8] Guibert N, Pradines A, Favre G, Mazieres J (2020). Current and future applications of liquid biopsy in nonsmall cell lung cancer from early to advanced stages. Eur Respir Rev.

[9] Giannopoulou L, Zavridou M, Kasimir-Bauer S, Lianidou ES (2019). Liquid biopsy in ovarian cancer: the potential of circulating miRNAs and exosomes. Transl Res.

[10] Cayrefourcq L, Alix-Panabières C (2020). Clinical relevance of liquid biopsy in breast cancer: update in 2020. Expert Rev Mol Diagn.

[11] Mazumder S, Datta S, Ray JG, Chaudhuri K, Chatterjee R (2019). Liquid biopsy: miRNA as a potential biomarker in oral cancer. Cancer Epidemiol.

[12] Moher D, Liberati A, Tetzlaff J, Altman DG; PRISMA Group (2009). Preferred reporting items for systematic reviews and meta-analyses: the PRISMA statement. BMJ.

[13] Chang YA, Weng SL, Yang SF, Chou CH, Huang WC, Tu SJ (2018). A Three-MicroRNA Signature as a Potential Biomarker for the Early Detection of Oral Cancer. Int J Mol Sci.

[14] Liu CJ, Tsai MM, Tu HF, Lui MT, Cheng HW, Lin SC (2013). miR-196a overexpression and miR-196a2 gene polymorphism are prognostic predictors of oral carcinoma. Ann Surg Oncol.

[15] Liu CJ, Lin JS, Cheng HW, Hsu YH, Cheng CY, Lin SC (2017). Plasma miR-187* is a potential biomarker for oral carcinoma. Clin Oral Investig.

[16] Liu CJ, Kao SY, Tu HF, Tsai MM, Chang KW, Lin SC (2010). Increase of microRNA miR-31 level in plasma could be a potential marker of oral cancer. Oral Dis.

[17] Lu YC, Chang JT, Huang YC, Huang CC, Chen WH, Lee LY (2015). Combined determination of circulating miR-196a and miR-196b levels produces high sensitivity and specificity for early detection of oral cancer. Clin Biochem.

[18] Mahmood N, Hanif M, Ahmed A, Jamal Q, Mushtaq S, Khan A (2019). Circulating miR-21 as a prognostic and predictive biomarker in oral squamous cell carcinoma. Pak J Med Sci.

[19] Pedersen NJ, Jensen DH, Lelkaitis G, Kiss K, Charabi BW, Ullum H (2018). MicroRNA-based classifiers for diagnosis of oral cavity squamous cell carcinoma in tissue and plasma. Oral Oncol.

[20] Sun G, Cao Y, Wang P, Song H, Bie T, Li M (2018). miR-200b-3p in plasma is a potential diagnostic biomarker in oral squamous cell carcinoma. Biomarkers.

[21] Tachibana H, Sho R, Takeda Y, Zhang X, Yoshida Y, Narimatsu H (2016). Circulating miR-223 in Oral Cancer: Its Potential as a Novel Diagnostic Biomarker and Therapeutic Target. PLoS One.

[22] Yang CC, Hung PS, Wang PW, Liu CJ, Chu TH, Cheng HW (2011). miR-181 as a putative biomarker for lymph-node metastasis of oral squamous cell carcinoma. J Oral Pathol Med.

[23] De Souza MG, de Jesus SF, Santos EM, Gomes ESB, de Paulo Santiago Filho A, Santos EMS (2020). Radiation Therapy Reduced Blood Levels of LDH, HIF-1α, and miR-210 in OSCC. Pathol Oncol Res.

[24] Emami N, Mohamadnia A, Mirzaei M, Bayat M, Mohammadi F, Bahrami N (2020). miR-155, miR-191, and miR-494 as diagnostic biomarkers for oral squamous cell carcinoma and the effects of Avastin on these biomarkers. J Korean Assoc Oral Maxillofac Surg.

[25] Ries J, Vairaktaris E, Agaimy A, Kintopp R, Baran C, Neukam FW (2014). miR-186, miR-3651 and miR-494: potential biomarkers for oral squamous cell carcinoma extracted from whole blood. Oncol Rep.

[26] Ries J, Baran C, Wehrhan F, Weber M, Neukam FW, Krautheim-Zenk A (2017). Prognostic significance of altered miRNA expression in whole blood of OSCC patients. Oncol Rep.

[27] Ries J, Baran C, Wehrhan F, Weber M, Motel C, Kesting M (2019). The altered expression levels of miR-186, miR-494 and miR-3651 in OSCC tissue vary from those of the whole blood of OSCC patients. Cancer Biomark.

[28] Chen L, Hu J, Pan L, Yin X, Wang Q, Chen H (2018). Diagnostic and prognostic value of serum miR-99a expression in oral squamous cell carcinoma. Cancer Biomark.

[29] Karimi A, Bahrami N, Sayedyahossein A, Derakhshan S (2020). Evaluation of circulating serum 3 types of microRNA as biomarkers of oral squamous cell carcinoma; A pilot study. J Oral Pathol Med.

[30] Li C, Feng Y, Shao W (2020). Changes of serum miR-223-3p in patients with oral cancer treated with TPF regimen and the prognosis. Oncol Lett.

[31] Maclellan SA, Lawson J, Baik J, Guillaud M, Poh CF, Garnis C (2012). Differential expression of miRNAs in the serum of patients with high-risk oral lesions. Cancer Med.

[32] Shi J, Bao X, Liu Z, Zhang Z, Chen W, Xu Q (2019). Serum miR-626 and miR-5100 are Promising Prognosis Predictors for Oral Squamous Cell Carcinoma. Theranostics.

[33] Singh P, Srivastava AN, Sharma R, Mateen S, Shukla B, Singh A (2018). Circulating MicroRNA-21 Expression as a Novel Serum Biomarker for Oral Sub-Mucous Fibrosis and Oral Squamous Cell Carcinoma. Asian Pac J Cancer Prev.

[34] Sun L, Liu L, Fu H, Wang Q, Shi Y (2016). Association of Decreased Expression of Serum miR-9 with Poor Prognosis of Oral Squamous Cell Carcinoma Patients. Med Sci Monit.

[35] Wen J, Xu H, Liu R, Chen Q, Dai Y, Xu Y (2020). MiR-92b as a marker for TPF induced chemotherapy response prediction and prognosis evaluation in with advanced oral squamous cell carcinoma patients. Cell Mol Biol (Noisy-le-grand).

[36] Xu H, Yang Y, Zhao H, Yang X, Luo Y, Ren Y (2016). Serum miR-483-5p: a novel diagnostic and prognostic biomarker for patients with oral squamous cell carcinoma. Tumour Biol.

[37] Dong G, Chen H, Shi Y, Jiang C, Yang H (2021). MicroRNA-758 Regulates Oral Squamous Cell Carcinoma via COX-2. Indian J Surg.

[38] Duz MB, Karatas OF, Guzel E, Turgut NF, Yilmaz M, Creighton CJ (2016). Identification of miR-139-5p as a saliva biomarker for tongue squamous cell carcinoma: a pilot study. Cell Oncol (Dordr).

[39] Gai C, Camussi F, Broccoletti R, Gambino A, Cabras M, Molinaro L (2018). Salivary extracellular vesicle-associated miRNAs as potential biomarkers in oral squamous cell carcinoma. BMC Cancer.

[40] He L, Ping F, Fan Z, Zhang C, Deng M, Cheng B (2020). Salivary exosomal miR-24-3p serves as a potential detective biomarker for oral squamous cell carcinoma screening. Biomed Pharmacother.

[41] Park NJ, Zhou H, Elashoff D, Henson BS, Kastratovic DA, Abemayor E (2009). Salivary microRNA: discovery, characterization, and clinical utility for oral cancer detection. Clin Cancer Res.

[42] Liu CJ, Lin SC, Yang CC, Cheng HW, Chang KW (2012). Exploiting salivary miR-31 as a clinical biomarker of oral squamous cell carcinoma. Head Neck.

[43] Mehdipour M, Shahidi M, Manifar S, Jafari S, Abbas FM, Barati M (2018). Diagnostic and prognostic relevance of salivary microRNA-21, -125a, -31 and -200a levels in patients with oral lichen planus - a short report. Cell Oncol.

[44] Momen-Heravi F, Trachtenberg AJ, Kuo WP, Cheng YS (2014). Genomewide Study of Salivary MicroRNAs for Detection of Oral Cancer. J Dent Res.

[45] Shahidi M, Jafari S, Barati M, Mahdipour M, Gholami MS Predictive value of salivary microRNA-320a, vascular endothelial growth factor receptor 2, CRP and IL-6 in Oral lichen planus progression. Inflammopharmacology. Inflammopharmacology.

[46] Zahran F, Ghalwash D, Shaker O, Al-Johani K, Scully C (2015). Salivary microRNAs in oral cancer. Oral Dis.

[47] Lima AM, Meira IA, Soares MS, Bonan PR, Mélo CB, Piagge CS (2021). Delay in diagnosis of oral cancer: a systematic review. Med Oral Patol Oral Cir Bucal.

[48] Irimie AI, Ciocan C, Gulei D, Mehterov N, Atanasov AG, Dudea D (2018). Current Insights into Oral Cancer Epigenetics. Int J Mol Sci.

[49] Alix-Panabières C, Pantel K (2021). Liquid Biopsy: From Discovery to Clinical Application. Cancer Discov.

[50] Osan C, Chira S, Nutu AM, Braicu C, Baciut M, Korban SS (2021). The Connection between MicroRNAs and Oral Cancer Pathogenesis: Emerging Biomarkers in Oral Cancer Management. Genes (Basel).

[51] Arantes LMRB, De Carvalho AC, Melendez ME, Lopes Carvalho A (2018). Serum, plasma and saliva biomarkers for head and neck cancer. Expert Rev Mol Diagn.

